# Early Life Interventions: Impact on Aging and Longevity

**DOI:** 10.14336/AD.202.0516

**Published:** 2025-07-05

**Authors:** Rong Yuan, Aida Adlimoghaddam, Yun Zhu, Xiuqi Han, Andrzej Bartke

**Affiliations:** ^1^Division of Geriatrics Research, Department of Internal Medicine, Southern Illinois University School of Medicine, Springfield, IL 62702, USA.; ^2^Department of Medical Microbiology, Immunology and Cell Biology, Southern Illinois University School of Medicine, Springfield, IL 62702, USA.; ^3^Department of Neurology, Center for Alzheimer's Research and Treatment, Neuroscience Institute, Southern Illinois University School of Medicine, Springfield, IL 62702, USA.; ^4^Department of Pharmacology, Southern Illinois University School of Medicine, Springfield, IL 62702, USA.

**Keywords:** Early life intervention, Mitochondria, Aging

## Abstract

Across mammals, lifespans vary remarkably, spanning over a hundredfold difference. Comparative studies consistently reveal a strong inverse relationship between developmental pace and lifespan, hinting at the potential for early-life interventions (ELIs) to influence aging and lifespan trajectories. Focusing on postnatal interventions in mice, this review explores how ELIs influence development, lifespan, and the underlying mechanisms. Previous ELI studies have employed a diverse array of approaches, including dietary modifications, manipulations of the somatotropic axis, and various chemical treatments. Notably, these interventions have demonstrated significant impacts on aging and lifespan in mice. The underlying mechanisms likely involve pathways related to mitochondrial function, mTOR and AMPK signaling, cellular senescence, and epigenetic alterations. Interestingly, ELI studies may serve as valuable models for investigating the complex regulatory mechanisms of development and aging, particularly regarding the interplay among somatic growth, sexual maturation, and lifespan. In addition, prior research has highlighted the intricacies of experimental design and data interpretation. Factors such as timing, sex-specific effects, administration methods, and animal husbandry practices must be carefully considered to ensure the reliability and reproducibility of results, as well as rigorous interpretation. Addressing these factors is essential for advancing our understanding of how development, aging, and lifespan are regulated, potentially opening avenues for interventions that promote healthy aging.

## Introduction

Mammalian evolution has produced a vast range of lifespans, varying over 100-fold. Comparative analyses consistently reveal a strong negative correlation between developmental pace and lifespan [1, 2]. The evolutionary theory of aging explains this consistent phenomenon as a trade-off, and suggests that antagonistic pleiotropic mechanisms are involved in regulating the relationship [3]. This association between development and aging, collectively termed "pace-of-life," provides a framework for understanding the complex interplay among development, including prenatal and postnatal development, and aging. Importantly, it implies that early life may present an important time window for interventions that can modify the aging trajectory and impact lifespan. This hypothesis is supported by the theory of developmental origins of adult health and disease (DOHAD), which originated from studying individuals whose mothers were exposed to starvation during pregnancy [4, 5]. The impact of nutritional stress, such as calorie restriction (CR) and low protein diets (LPD), during prenatal development on adult health was subsequently shown in numerous studies in laboratory rodents and domestic sheep [6, 7]. These studies indicated that maternal diet alterations during pregnancy and/or lactation can have significant impacts on postnatal growth and metabolism [6]. Additionally, adult pathological characteristics, including the risk of obesity and chronic noninfectious diseases, also are shaped by events during early postnatal development, childhood, and adolescence. Previous studies revealed that adverse childhood experiences including abuse, famine, and lower socioeconomic position are related to unfavorable body composition, cardiovascular disease, type 2 diabetes, and other health problems in various human populations [8-17]. Importantly, early-life factors have been related to the risk of multimorbidity [18-20]. The rise in multimorbidity suggests that a factor influencing the risk of various diseases mediates the effects of early-life adversity. According to the “geroscience” concept [21], accelerated aging could well represent the mechanistic link between adverse childhood events and adult health.

Studies in mice provide compelling evidence supporting this hypothesis. For instance, it was shown that selecting mice for slower body weight growth during the postnatal period (0-10 days) correlates with significantly increased lifespan [22]. Utilizing mouse models and treatments administered during early postnatal life, studies have further explored the influence of early-life events on aging and lifespan. This review focuses specifically on postnatal early-life interventions (ELIs) in mice, discusses the impact of ELIs on development and lifespan, provides insights into potential interventions in humans, and elucidates the underlying mechanisms. It will begin by examining previous ELIs such as diet modifications, hormone therapies, and chemical treatments on aging and lifespan in mouse models. Subsequently, it will address potential factors influencing the impact of ELIs and the considerations in designing ELI mouse studies.

## ELIs exhibit notable effects on aging and lifespan in mice.

### Diet modifications

Calorie restriction (CR), a reduction in calorie intake without malnutrition, is a well-established intervention shown to extend lifespan in various organisms. Extensive research uncovered the complex mechanisms underlying the anti-aging effects of CR, including delayed cellular senescence, optimized mitochondrial function, improved autophagy, and less chronic inflammation. These changes reduce the risk and slow the progression of age-related diseases and frailty, leading to an extended healthspan and lifespan. Ozanne S.E., *et al.* found that restricting the growth of male mouse pups during the neonatal period (from day 1 to day 21) by feeding dams a low-protein diet (8% protein) not only extended longevity, but also protected against the life-shortening effects of an obesity-inducing diet in later life, compared to male pups whose mothers were fed a diet containing 20% protein [23, 24]. Conversely, in studies of pregnant dams fed low-protein diet during pregnancy but switched to a diet containing 20% protein during lactation, the accelerated compensatory postnatal growth to make up for restricted in-utero growth significantly reduced lifespan of the offspring and had a detrimental impact on longevity associated with obesity-inducing diet after weaning.

Another study, using genetically heterogeneous UM-HET3 mice, investigated the impact of dietary ELIs on lifespan by modifying maternal diets (20% vs. 8% protein diet) and manipulating access to diet by enlarging litter size (8 vs. 12 pups; aka “crowded litter”) [25]. Combining data from mice of both sexes, those in the crowded litter group had prolonged median survival in comparison to controls (874 days versus 740 days). This effect was especially notable in females, where the difference was statistically significant (848 days versus 731 days), but not in males. Mice whose mothers were fed a low-protein diet during lactation did not exhibit significant differences in survival. Although not entirely aligned, the results of these studies highlight the potential for extending lifespan and enhancing health by restricting growth during early life [25].

### Somatotropic axis interventions

The GH/IGF1 pathway is a well-recognized regulator of lifespan across diverse species, ranging from worms to mammals. Numerous studies have consistently shown that suppressing this pathway effectively inhibits cancer development [26, 27]. Remarkably, mutations resulting in the downregulation of this pathway, as observed in Ames and Snell dwarf mice, reduce body mass and size, decrease reproductive capacity, improve metabolic function during aging, and notably extend lifespan [28]. These observations provide a prime example supporting the evolutionary theory of antagonistic pleiotropic genes, which proposes a trade-off relationship between prioritizing early development and delaying aging processes. Administering GH treatment to dwarf mice at an early age offers a valuable model for investigating this trade-off relationship. In male Ames dwarf mice, GH subcutaneous injection (6 µg/g bw/d, given in equally divided doses 2x/d) from day 15 to day 56 significantly reduced lifespan [29]. Subsequent studies confirmed this effect in both female and male Ames dwarf mice [30]. Interestingly, the lifespan of dwarf mice treated once daily with a dosage of 4 µg/g bw/d remained unchanged, suggesting that the detrimental effect on lifespan may depend on the dosage and frequency of administration [29]. Importantly, the transient ELI that shortened lifespan also significantly altered the metabolic profile at old age (20 months), including elevated circulating glucose and insulin along with reduced adiponectin [30]. The treatment also had long-lasting effects at old age, including increased inflammation in the liver, white adipose tissue, and hypothalamus [31]. Intriguingly, dermal fibroblasts from Ames dwarf mice display increased resistance to the cytotoxic effects of cadmium, paraquat, methyl methanesulfonate, and rotenone-induced cell death compared to fibroblasts from wild-type controls. However, this enhanced stress-resistance phenotype is abrogated by early-life GH treatment [31, 32]. These results suggest that the reduced lifespan observed with employing GH as an ELI may be associated with impaired cellular stress response mechanisms, potentially contributing to heightened susceptibility to age-related damage and oxidative stress.

Furthermore, using GH as an ELI in Ames dwarf mice also sheds light on the regulatory mechanisms of GH in the central nervous system (CNS) in regard to metabolism. Dwarf mice exhibit a reduction in the development of both appetite-stimulating AgRP neurons in the arcuate nucleus (ARH) and appetite-suppressing POMC neurons throughout the hypothalamus, reducing their projections to key regions like the paraventricular nucleus (PVH) and dorsomedial nucleus (DMH), consistent with a critical role for GH signaling in regulating appetite and energy balance within the hypothalamus [33]. Notably, GH treatment as an ELI reversed these effects in Ames dwarf mice, even when examined at 18 months, demonstrating long-lasting influence [33]. Interestingly, disrupting the GH receptor specifically in the liver, which reduced circulating IGF-1 without lifespan extension, did not affect AgRP and POMC neurons [33]. This implies that GH itself, rather than the downstream factor IGF-1, is essential for hypothalamic control of food intake and energy balance. These results highlight the potential of using a transient ELI targeting GH signaling to induce long-lasting changes in the CNS, ultimately influencing healthspan and lifespan.

While the precise molecular mechanisms underlying the long-lasting effects of GH as an ELI are still under investigation, emerging evidence points towards the involvement of epigenetic mechanisms, particularly those involving histone H3 modifications. Histone H3 is a critical component of chromatin, the complex of DNA and proteins that forms chromosomes. Post-translational modifications of histone H3, such as methylation and acetylation at specific lysine residues, play key roles in regulating gene expression by influencing chromatin structure and accessibility. Studies in GH-deficient Ames dwarf mice revealed suppressed levels of H3K4me (methylation at lysine 4) in hepatic and brain tissues, alongside elevated levels of H3K27me (methylation at lysine 27) in the brain. GH as an ELI has been shown to significantly alter histone H3 markers in these tissues [34]. Furthermore, GH as an ELI has been associated with increased acetylation of histone H3 at various lysine residues, such as H3K14ac (acetylation at lysine 14), H3K18ac (acetylation at lysine 18), and H3K27ac (acetylation at lysine 27), with the changes being tissue-specific [34]. These findings suggest that histone H3 modifications play a role in mediating the long-term effects of GH as an ELI on aging and lifespan through epigenetic regulation.

## Chemical treatments: Key regulatory pathways like

AMP-activated protein kinase (AMPK) and the mammalian target of rapamycin (mTOR), crucial regulators of metabolism, have emerged as major pathways for regulating development and aging. AMPK acts as a cellular energy sensor, promoting energy production and downregulating energy-consuming processes during low-energy states [35]. Conversely, mTOR promotes anabolic processes when nutrients are abundant [36]. These opposing roles offer insights into how metabolism is regulated and how these processes potentially impact development and aging. Interestingly, both AMPK activation and mTOR inhibition share downstream cellular effects such as stimulating autophagy, promoting mitochondrial biogenesis, suppressing inflammation, and delaying cellular senescence, potentially contributing to a delay in age-related diseases [36, 37].

**A. ELIs targeting mTOR**: The mTOR signaling pathway involves two distinct complexes: mTORC1 and mTORC2. Often referred to as the "growth regulator," mTORC1 integrates diverse signals such as amino acids, growth factors, and oxygen levels to modulate cellular responses. mTORC1 activation triggers enhanced protein translation, ribosome biogenesis, and lipogenesis, concurrently inhibiting the catabolic process of autophagy [38]. mTORC2, known as the "survival complex," is activated by various factors including growth factors and insulin signaling through PI3K activity. It plays a role in regulating cytoskeletal organization, cell survival, and metabolism, specifically through Akt activation [39]. However, the influence of mTORC2 on metabolism is less well-defined than mTORC1. Importantly, these complexes exhibit crosstalk, influencing each other's activity and downstream signaling pathways [38, 39].

The mTOR pathway plays a pivotal role in regulating the trade-off relationship between development and aging [40]. In early life, mTOR signaling promotes development, driving accelerated growth, sexual maturation, and increased reproductive capacity. However, mTORC1 activity in later life appears detrimental, contributing to age-related pathologies including neurodegeneration, metabolic dysfunction, and cancer [40-44]. Importantly, in laboratory mice, suppressing mTOR activity in adulthood from middle age to old age shows potential of extending lifespan and promoting healthy aging [40-48]. These benefits are attributed to various mechanisms, including delayed cellular senescence, reduced chronic inflammation, enhanced autophagic clearance, and improved mitochondrial function [40-47, 49-51].

Given the antagonistic effects of mTOR signaling in development and aging, evolutionary aging theory suggests that ELIs targeting this pathway might hold promise for extending healthspan and lifespan. Supporting this hypothesis, recent studies in diverse mouse models reported compelling results [52, 53]. In CD1 mice, an outbred population, *i.p.* injections of rapamycin, between postnatal days (PND) four and 30, led to reduced body/organ size and significantly extended lifespan in both sexes compared to controls. Similarly, UM-HET3 pups receiving an ELI (PND 0-45) *via* rapamycin-supplemented maternal diet also displayed persistent reductions in body size across their lifespan, with treated males also exhibiting significantly increased lifespan. Interestingly, regardless of administration methods, both models showed improved healthspan *via* frailty index assessment. Moreover, the observed lifespan extension transcends species, evident in studies of *Drosophila melanogaster* and *Daphnia magna*, suggesting an evolutionarily conserved role of the mTOR pathway in regulating lifespan [52, 53]. These findings underscore the potential of ELIs targeting the mTOR pathway as a promising strategy for extension of lifespan and healthspan.

**B. ELIs targeting AMPK**: AMPK monitors the balance between energy-rich ATP and its breakdown product, AMP. When energy levels are reduced, the rising AMP:ATP ratio activates AMPK, prompting changes to restore balance. Metformin, a cornerstone of diabetes management, activates AMPK, a cellular energy sensor. This activation promotes energy availability by increasing glucose uptake and fatty acid oxidation, while simultaneously suppressing energy-intensive processes like protein and lipid synthesis. Ultimately, AMPK helps maintain cellular energy homeostasis, contributing to the glucose-lowering effects of metformin. Metformin has shown significant effects in attenuating aging processes. It protects against macromolecular damage, delays stem cell aging, and mitigates telomere attrition and senescence, partly through its activation of AMPK and subsequent metabolic changes [54-56].

Exposure to metformin during lactation has been found to influence metabolism in young adults [57]. In a study utilizing inbred C57BL/6 (B6) mice, dams were administered metformin *via* drinking water (3mg/mL metformin-HCl) from birth to PND 21. Offspring from the metformin-treated group, regardless of sex, exhibited a leaner phenotype with a higher proportion of small adipocytes in gonadal white adipose tissue. Male, but not female, offspring demonstrated improved glucose tolerance at two months, accompanied by a slight increase in insulin secretion in response to glucose *in vivo*. In response to a high-fat diet, female offspring displayed protection against weight gain compared to controls, alongside mild enhancements in glucose tolerance, increased lean mass, and decreased fat mass. In contrast, male offspring exhibited protection against insulin resistance, without alterations in lean or fat mass, compared to controls [57]. These findings underscore the differential impacts of lactational metformin exposure on metabolic health between male and female offspring, suggesting its potential as a protective factor against metabolic disorders, albeit with sex-specific nuances.

Regarding the impact of metformin on lifespan, previous experiments in mice have shown inconsistent results. For example, in male B6 and B6C3F1 mice, low-dose metformin (1000 ppm in the drinking water) increased lifespan when started at 12 months of age, while a higher dose (10,000 ppm in the drinking water) decreased lifespan [56]. An NIH-sponsored Intervention Testing Program (ITP) study found no significant impact on longevity when metformin treatment began at the age of 10 months in UM-HET3 mice [58]. However, the addition of metformin (1000 ppm) to rapamycin (14 ppm) in the diet extended lifespan compared to historical cohorts treated with rapamycin alone, suggesting potential additive benefits with co-treatments [56]. Additionally, metformin treatment during early life showed effects on lifespan in a strain- and sex-specific manner. For instance, neonatal 129/Sv pups treated with metformin at days three, five, and seven (i.p. 100 mg/kg) exhibited a significant increase in the lifespan of males but not females [59]. Similarly, when started at the age of three months in outbred Swiss H Rappolovo mice, metformin treatment significantly increased the lifespan of female mice [60]. Focusing on the short-term effects on development and metabolism, metformin as an ELI (PND 15 to 56, *i.p.* xx mg/kg) was tested in B6 and UM-HET3 mice [61, 62]. In B6 mice, the treatment did not significantly alter somatic growth, assessed by level of circulating IGF1, body weight, and body size, or alter onset of female or male puberty, measured by the ages of vaginal opening and prepuce separation, respectively. The treatment significantly improved glucose tolerance in B6 males, but not females, and both female and male mice exhibited impaired insulin tolerance [61]. Surprisingly, in the heterogenous UM-HET3 mice, the same treatment significantly increased body weight and food consumption in both female and male pups. Tail length was longer and circulating IGF1 levels were elevated in both sexes, indicating a non-sex-specific enhancement of somatic growth. Importantly, the treatment had sex-specific impacts on puberty onset. A significant delay in the age of vaginal patency was observed in females, while males did not exhibit a significant alteration in the age of prepuce separation. Additionally, the ELI significantly improved insulin sensitivity in the UM-HET3 female mice, measured by quantitative insulin sensitivity check index (QUICKI), but had the opposite effect in male pups [62]. These findings indicate that the genetic background, sex, and age of treatment initiation play important roles in regulating the effects of metformin on development, aging, and lifespan. However, the underlying mechanisms have not been adequately investigated.

**Potential role of mitochondrial function in the long-term effects of ELIs on aging:** Mitochondrial dysfunction can lead to a range of health problems, including aging, and many other age-related disorders such as neurodegenerative diseases, cardiovascular issues, and metabolic disorders [63]. While direct investigations into the long-term effects of ELIs on mitochondrial function are lacking, extensive research on diet restriction, treatment with metformin and rapamycin, and GH/IGF-1 interventions suggest a potential role for ELIs in regulating mitochondrial function, thus influencing aging and lifespan. For instance, low protein and caloric restriction diets can improve mitochondrial function *via* interference with dynamics (i.e., fusion and fission), respiration, and related oxidative stress [64]. The GH-IGF-1 axis plays a complex and sometimes contradictory role in mitochondrial function. While GH may increase mitochondrial workload due to its growth-promoting effects, the overall impact appears multifaceted. Studies suggest that reduced GH/IGF-1 signaling in long-lived mice leads to adaptations for efficient energy production, with higher oxygen consumption and potentially increased activity of complex IV, a key enzyme in the electron transport chain [65-67]. Conversely, increased GH/IGF-1 signaling in short-lived mice might indicate inefficient energy use, as shown by decreased oxygen consumption [68]. However, IGF-1 itself seems crucial for maintaining healthy mitochondria. Mice with reduced IGF-1 levels exhibit elevated ROS and impaired function, while IGF-1 treatment in mice and aged rats improves mitochondrial health by restoring membrane potential, oxygen consumption, and ATP production [69]. The mechanisms underlying the effects of these interventions on mitochondrial function involve the regulation of the AMPK and mTOR signaling pathways. Targeting these pathways, metformin and rapamycin exert multifaceted effects on mitochondria and contribute to an overall healthier mitochondrial population. The benefits of metformin likely involve reduced oxygen consumption and activation of AMPK signaling, leading to improved function, reduced stress, and enhanced biogenesis [70]. Rapamycin, on the other hand, influences mitochondrial health by inhibiting mTOR signaling, potentially promoting new mitochondrial generation and triggering mitophagy, a selective process that eliminates dysfunctional mitochondria [71-74].

Evidence from previous studies strongly suggests that early-life stress causes adverse effects on health at older age *via* the impacts on mitochondrial function. For instance, mtDNA is reduced in individuals with childhood adversity [75, 76]. mtDNA contains genetic instructions necessary for the synthesis of proteins that are crucial in cellular respiration and other energy-producing functions. Therefore, adverse experiences in early life may impact mitochondrial function, potentially affecting overall energy metabolism and cellular processes. Further strengthening this link, recent studies indicate that adults with a history of adverse childhood experiences exhibit compromised ATP production and mitochondrial respiration in muscle tissue, which may be involved in increasing the susceptibility to metabolic disorders [77, 78]. Notably, early-life stress modifies gene expression related to mitochondrial fission, and these changes persist into adulthood, influencing mitophagy in muscle tissue and fission pathways in the hippocampus [78]. Collectively, combining evidence of the long-term effects of ELIs on healthspan and lifespan with the established impacts of dietary restriction, GH/IGF1 intervention, and treatments with rapamycin and metformin on mitochondrial health and function, it will be intriguing to explore how these ELIs could contribute to long-term benefits on mitochondrial function, potentially playing a pivotal role in extending lifespan. This presents a clear knowledge gap that needs to be filled, highlighting the necessity for further research in this area.

## ELIs provide a variety of models for investigating the complicated regulatory mechanisms of development and aging.

Numerous studies suggest that somatic growth and sexual maturation, both critical developmental processes, are tightly co-regulated. In aging studies, anti-aging models like dwarf mice and dietary restriction across various species exemplify this connection [79-82]. Demonstrating the co-regulation, several ELIs, including maternal protein restriction, rapamycin treatment, and stress (reduced nesting material) can decrease body weight and delay sexual maturation in rodents [83-85], providing valuable models to explore the co-regulatory mechanisms.

Somatic growth and reproductive maturation are each fueled by a continuous influx of energy. Interestingly, these seemingly co-regulated processes are governed by distinct hormonal axes. Reproductive maturation is orchestrated by the gonadotropic axis, initiated in the hypothalamus by kisspeptin (Kiss1), and involves a cascade of hormones and receptors (GnRH, GnRHR, LH, FSH). Somatic growth is controlled by the growth hormone (GH) axis, with GH and its signaling pathway (GHRH, GHRHR, GHR, IGF-1) playing a leading role. This intriguing compartmentalization raises questions. Given their shared energy demands, do these resource-intensive processes compete for limited nutrient and energy reserves? Furthermore, can these axes be manipulated to regulate growth and reproduction independently? Exploring the potential for decoupling these processes holds significant implications for understanding development and potentially influencing healthspan and lifespan.

Previous studies support the hypothesis that the regulation of somatic growth and sexual maturation could be decoupled. In the 1980s, L.C. Drickamer conducted a series of genetic studies investigating female sexual maturation rates in mice. Using outbred mice derived from the ICR strain, Drickamer successfully selected lines with distinct phenotypes: females exhibiting fast and slow sexual maturation [86]. Subsequent reverse selection - breeding for slow maturation from the fast line and vice versa - demonstrated a remarkable ability to reverse the established phenotypes [87]. This suggests a strong genetic influence on the timing of sexual maturation. An important finding of this research was the observed dissociation between sexual maturation and somatic growth. When reversing the selection for maturation rate, pup body weight remained largely unchanged [87]. This indicates that these traits can be genetically manipulated independently, highlighting the potential for separate regulatory mechanisms. Furthermore, studies in transgenic mice also support the hypothesis. For instance, female transgenic mice were created to overexpress similar levels of either intact insulin-like growth factor-binding protein-2 (IGFBP-2) (D-mice) or a mutant IGFBP-2 lacking the Arg-Gly-Asp (RGD) motif (E-mice) [88]. Both groups displayed comparable growth impairment (-9% and -10%) compared to wild-type controls (C-mice). However, D-mice exhibited a crucial difference: delayed sexual maturation and increased lifespan, presumably related to reduced bio-availability of IGF-1. In contrast, E-mice showed no alterations in sexual maturation or lifespan. These findings highlight the dissociation between reproductive maturation and somatic growth. Importantly, they suggest a less direct link between somatic growth and lifespan compared to reproductive maturation [88].

ELI studies also suggest that the regulation of somatic growth and reproductive maturation can be decoupled. For instance, it is well-known that exposing pups to the odor of adults of the opposite sex promotes sexual maturation; however, the alterations in somatic growth are dependent on the nature of the odor and the genetic background. Exposing female pups to adult female odor has been shown to delay the age of sexual maturation, along with increasing lifespan, but does not significantly alter body weight [89-93]. Interestingly, metformin treatment in UM-HET3 pups resulted in increased body weight, extended tail length, and elevated circulating IGF1 levels, but with a delayed age of vaginal opening [62], thus presenting a model in which sexual maturation and somatic growth are regulated oppositely. These treatments present experimental models to investigate whether suppressing female reproduction, other than somatic growth, is directly linked to extended lifespan, and could offer novel insights into the molecular mechanisms regulating the tradeoff relationship between reproductive development and aging. Additionally, in considering translation to humans, ELIs that do not reduce stature could enhance public acceptability.

While the precise mechanism remains unclear, the dissociation between somatic and gonadotropic development in UM-HET3 pups exposed to metformin as an ELI may result from AMPK activation signaling an energy deficit, subsequently suppressing gonadotropic development [94-96]. Intriguingly, in metformin-treated mice that consumed more food than controls, ample food supplementation appeared to shift nutrient allocation towards somatic growth, evidenced by an upregulated circulating IGF1 level, increased body weight, and enlarged body size [62]. This potential for decoupling somatic growth and reproductive maturation warrants further investigation. Notably, metformin-induced decoupling may be genetically dependent. This is evidenced by different outcomes observed in B6 female pups, where the ELI of metformin treatment significantly reduced body weight, but did not affect age of sexual maturation. This observation necessitates further verification in diverse mouse models and other species.

## Unique factors need to be considered in designing ELI mouse studies.

In addition to standard considerations for aging research using mice, such as determining sample sizes for lifespan studies and establishing control and experimental groups, the nature of ELIs necessitates specific experimental design considerations. These include determining the timing and method of treatment administration, considering sex differences, and ensuring specific husbandry conditions meet experimental requirements.

**Time window-specific effects of ELIs**: Building on the compelling evidence for altered lifespan and healthspan by ELIs of diet modifications, chemical treatments, or hormone therapies, previous experiments strongly suggested that a critical treatment time window exists for regulating aging and lifespan. For instance, the Prop1 mutation in Snell dwarf mice causes anterior pituitary deficiency, leading to suppressed growth hormone (GH) secretion. This deficiency contributes to delayed development and is associated with extended lifespan, similar to that observed in Ames dwarf mice [97]. In Snell dwarf mice, treatment with GH plus thyroxine, initiated at the age of four weeks and spanning 11 weeks, resulted in increased body weight and reinstated fertility in males; however, no significant alteration in lifespan was observed. In contrast to the aforementioned experiments, GH treatment initiated at earlier ages (1 or 2 weeks) in Ames dwarf mice, significantly reduced lifespan and compromised resistance to aging-related frailties [30-32]. These variances suggest that the timing of the treatment likely accounts for the observed potential impacts. The sensitivity to timing is further exemplified by the experiments of rapamycin as an ELI. For example, the study of rapamycin-treated CD1 mice investigated long-term effects on healthspan and lifespan in two different ELI time windows, PND 4-30 *vs.* PND 30-60 [53]. The results showed that with a similar extension of the inhibition in mTOR activity, the PND 4-30 treated mice displayed significantly longer lifespans and improved frailty index compared to mice treated during PND 30-60, which showed no significant differences in lifespan compared with the control group. This intriguing difference suggests that the effective window for delaying aging in this study lies before PND 30. Interestingly, a previous study reported that the average age of vaginal patency in CD1 mice is 31.3 days [98]. While husbandry conditions can influence the exact timing of sexual maturation, this observed coincidence raises the possibility of a link between the long-term lifespan effects of ELIs and the time window coinciding with the onset of reproductive maturity.

**Figure 1.**
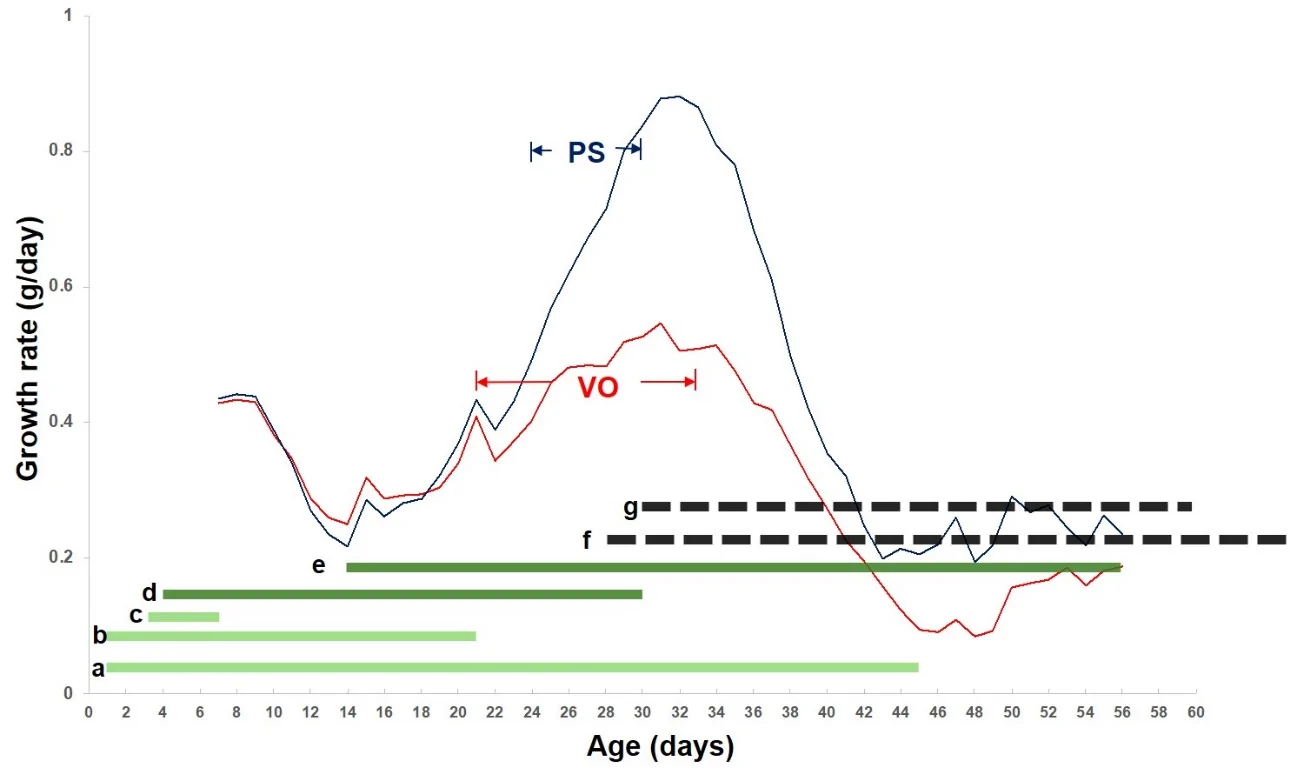
**Potential impact of the time window on early-life intervention effects on lifespan: The curves represent the 7-day rolling average growth rates (g/day) of female (red) and male (blue) UM-HET3 pups [[62] and unpublished data]**. Previous studies on the effects of ELIs are shown for reference (a-g) [23, 25, 30, 52, 53, 59]. Lighter and dark green bars indicate longevity alterations observed in one sex or both sexes. Black dot bars indicate no significant change in lifespan. a. Rapamycin treatment, days 1 to 45, increased the longevity of male UM-HET3 mice. b. CR in the first 3 weeks extended the lifespan of male UM-HET3 mice. c. Metformin treatment at day 3, 5, 7 extended lifespans of 129S males. d. Rapamycin treatment, day 4 to 30, significantly extended the lifespan of male and female CD1 mice. e. GH treatment, day 14 to 56, significantly reduced the lifespan of female and male AD mice. f. GH treatment, day 28 to 105, did not alter the lifespan of Snell dwarf mice. g. Rapamycin treatment, day 30 to 60, did not alter the lifespan of CD1 mice. The VO and PS of UM-HET3 pups, occur between day 21 to 43 and day 24 to 30 [62].

It is crucial to note that ELIs in mice, which typically last from several days to a few weeks, are generally seen as transient. However, when a treatment in mice extends from birth to approximately 35 days, coinciding with the age of female sexual maturation in most laboratory mice [99], extrapolating this timeframe to humans implies treatment from birth to around 12 years old—the age of menarche for many girls in the United States [100]. This length of treatment cannot be considered transient, and in consideration of translation to humans, is less likely to be widely accepted. Therefore, investigating the effects of development stage-specific ELIs on aging is crucial for developing translational methods and probing underlying molecular mechanisms. The Figure 1 depicts developmental traits, including body weight gain (g/day) and ages of sexual maturation (indicated by vaginal opening or preputial separation) of UM-HET3 pups that were raised in our animal facility under regular conditions ([62] and unpublished data). Embedded bars represent the time windows of ELIs in mouse models and their impact on lifespan. Despite variations in developmental pace among mouse populations and husbandry conditions, this figure raises the hypothesis that ELIs may exert time window-specific effects on lifespan, which should be considered in the design of future experiments.

**2. Sex-specific effects of ELIs:** While rapamycin administered *via i.p.* injection in CD1 mice between PND 4 and 30 had a sex-independent effect on lifespan [53], similar to what has been seen with rapamycin treatment at older ages [45-47], sex-specific effects have been observed when UM-HET3 pups were exposed to dietary supplementation of rapamycin for the initial 45 days of life [52]. In this scenario, the lifespan of male, but not female, mice was significantly extended. In addition, sex-specific effects of ELIs are also evident in responses to hormonal interventions. In Ames dwarf mice, GH treatment between two and eight weeks of age reduced the lifespan of both sexes. However, a different outcome occurred when GH treatment was administered between one and seven postnatal weeks - only the male dwarf lifespan was significantly reduced. No significant alteration in female lifespan was observed. These findings suggest that not only the timing of ELIs impacts the effects, but also the interaction between sex and the timing of treatment. Further evidence comes from a UM-HET3 diet restriction study. Enlarging litter size (12 vs. 8 pups/dam) significantly increased female lifespan, but had no significant impact on male lifespan [25].

The sex difference in response to ELIs is also evidenced by studies of olfactory cues. It has been previously established that rodent pups exposed to the olfactory cues of adults had significantly altered age of sexual maturation [101-106]. Specifically, female mice exposed to male odors (soiled bedding or urine) during development experience earlier sexual maturity. Conversely, exposure to odors from group-housed adult females can delay sexual development. Similarly, exposing males to females or their odors has been associated with increased testes and seminal vesicle size early in life, suggesting accelerated sexual maturation [107]. These odor-based priming effects are believed to be adaptive strategies [101-107]. Animals can adjust the timing of early-life reproduction based on anticipated future environments. Interestingly, the alterations in female sexual maturation are associated with changes in lifespan. For instance, it has been shown female mice with earlier sexual maturity experienced increased mortality within the first 180 days of life and have reduced litter sizes [108]. Furthermore, exposure to female adult odor cues during early life can significantly extend the lifespan of female mice [89]. However, no lifespan alteration was observed in male mice when exposed to either female or male adult odor cues [89]. These findings suggest that the co-regulation of sexual maturation and lifespan observed in female mice may not extend to males, emphasizing the existence of sex-specific mechanisms governing lifespan.

The human female lifespan advantage is a well-documented phenomenon observed across diverse environments [109]. However, this advantage is not consistently replicated in traditional rodent models. An analysis of numerous mouse survival studies revealed no consistent sex difference in lifespan [110]. Similarly, analysis of lifespan in 31 inbred mouse strains revealed no overall sex difference in *Mus musculus* [111]. The absence of a female lifespan advantage in traditional mouse models poses a significant challenge for investigating the mechanisms of human longevity and designing interventions. However, UM-HET3 mice present a potential avenue for addressing this limitation. These mice are a genetically heterogeneous population, produced by crossing four inbred strains, and were created to mimic the complexity of the human genome [112]. Importantly, UM-HET3 mice exhibit a female lifespan advantage similar to humans, with the greatest difference observed in early adulthood [113]. Furthermore, UM-HET3 mice provide valuable insights into sex-specific factors influencing longevity. There is a stronger inverse relationship between bodyweight and lifespan in males compared to females [113]. Additionally, male survival exhibits greater variation across environments, suggesting a higher female resilience to environmental factors influencing survival. Interestingly, the relationship between body weight and lifespan in both sexes mirrors the human condition, shifting from negative to positive in later life [113]. These unique features make UM-HET3 mice a highly suitable model for ELI studies on aging and lifespan. Their female-specific lifespan advantage, coupled with insights into bodyweight influences, enables researchers to investigate sex-specific mechanisms underlying aging and develop targeted interventions for both sexes.

Although *Mus musculus* may not exhibit robust sex disparities overall, analysis of the individual mouse strains shows that a few strains do exhibit differences in longevity between sexes. Among 31 inbred strains, combining log-rank tests, comparisons of median and maximum lifespan found that males have greater longevity in three strains (129S1, NOD, and NZW), while female B10 and P mice have a significantly greater lifespan than males [111]. Due to sex-specific longevity observed in the 129S lineage, which is a common source of embryonic stem cells for transgenic mouse models, accounting for sex as a biological variable is essential when designing experiments and interpreting data from ELI studies utilizing these models. Furthermore, sex differences in lifespan can sometimes reflect underlying pathological variations. For example, NOD mice, a common model for diabetes research, exhibit sex-based susceptibility. Female NOD mice are significantly more prone to autoimmune diabetes compared to males [114], and studies showed androgen treatment can lessen this risk in females [115]. This suggests a potential link between sex-specific susceptibility to certain diseases and lifespan regulation. Therefore, incorporating sex as a variable is crucial when designing ELI studies using NOD mice to investigate diabetes and lifespan. Disregarding sex disparity in this model could lead to skewed results.

**Influences of administration approaches**: According to the methods of administration, ELIs can be categorized into two groups: maternal and offspring administration.

**A. Maternal administration**: Maternal diet intervention may impact offspring both directly and indirectly. Direct effects can occur through the delivery of treatment to offspring *via* milk. For instance, rapamycin can be detected in milk following the administration of rapamycin to dams. Therefore, rapamycin can influence offspring development directly [52, 53]. Furthermore, maternal treatment with rapamycin can alter the protein and lipid contents in milk and its production, thereby indirectly impacting offspring [116, 117]. Within the lactating mammary gland, specific amino acids (AAs) act as messengers, informing milk production based on the metabolic state [117]. Among these regulators, mTORC1 integrates signals from cellular stressors, growth factors, and nutrients to control key processes like protein and fat synthesis, as well as protein breakdown (autophagy). A clear link between mTORC1 activity and casein synthesis was detected in both lab animals and animals in the natural environment, with AAs acting as activators [118]. The suppression of mTOR by rapamycin can lead to a reduction in milk production, as well as reduced concentrations of proteins and fat content in the milk. For instance, treating lactating B6 dams with rapamycin significantly inhibited mTORC1 activity in the mammary glands, resulting in reduced milk production and a decrease in body weights of the pups, with rapamycin detected in the livers of the offspring [118]. Similarly, activation of AMPK *via* metformin treatment or diet restriction has been reported to significantly inhibit milk fat and protein synthesis. The underlying mechanism may involve the regulation of prolactin signaling by triggering prolactin receptor (PrlR) ubiquitination via β-transducin repeat-containing protein (β-TrCP) regulation. This would lead to PrlR degradation through lysosomal endocytosis and subsequent attenuation of prolactin signaling [119]. Furthermore, AMPK activation can inhibit milk fat synthesis through two main mechanisms: suppressing the generation of new fatty acids (de novo fatty acid synthesis) and promoting the breakdown of existing fats (fatty acid oxidation). This regulation occurs via changes in key enzymes and transcriptional factors, along with a reduction in the acetylation of peroxisome proliferator-activated receptor gamma coactivator-1 alpha (PGC-1α) [119]. Considering the key role of milk production and composition in offspring development, it is essential to account for the effects on milk when designing ELI experiments and interpreting their outcomes.

**B. Offspring administration**: Intraperitoneal (*i.p.*) route of drug administration in rodents is a widely used approach that delivers the treatment directly to the offspring. It is considered as a justifiable route for pharmacological and proof-of-concept studies to evaluate the effect(s) of target engagement [120]. It offers precise dosing, enabling accurate drug delivery, and rapid absorption into the bloodstream. Moreover, it is generally perceived as straightforward to administer, efficient, suitable for chronic treatments, and associated with low levels of stress [120]. However, the risk of complications such as infection or peritonitis exists if not performed correctly. In addition, a major concern of *i.p.* is that the route of administration can influence drug effects, particularly concerning the microbiome. For example, metformin may modulate microbiota, suggesting potential differences in regulatory effects between oral and *i.p.* administration [121]. Furthermore, conducting *i.p.* injections demand careful consideration of solvent selection due to its potential impact on drug solubility, absorption kinetics, pain, and overall study outcomes. While ethanol offers good solubility for a broad range of compounds and facilitates rapid absorption, its irritant properties may cause tissue irritation upon injection, potentially confounding study results. Moreover, the behavioral effects and toxicity of ethanol at high concentrations warrant caution in experimental design and data interpretation. For instance, *i.p.* injection of rapamycin, dissolved in ethanol, significantly increased lifespan in the group treated from PND 4 to 30 [53]. However, it should be noted that this method results in exposing young mice to ethanol levels equivalent to heavy drinking in adult humans, raising concerns about the impact of ethanol on the outcome [122]. Specifically, the rapamycin/ethanol solution elevated the expression of sulfotransferases in liver [53]. Sulfonation, a crucial metabolic reaction, plays a key role in processing various xenobiotics, drugs, and endogenous compounds. Alcohol sulfotransferase catalyzes the sulfate conjugation of primary and secondary alcohols [123]. Instead of solely altering the natural aging trajectory through rapamycin administration, the observed lifespan extension may be linked to the protective effects of concurrent upregulation of sulfotransferases, as compared to control mice that also received ethanol but no rapamycin treatment. Saline, a physiologically compatible solvent, minimizes adverse effects on animals and is generally well-tolerated. However, using saline may present challenges for certain drugs with poor solubility. Dimethyl sulfoxide (DMSO) stands out as a versatile solvent capable of dissolving a wide range of compounds and enhancing drug distribution. However, its toxicity at high concentrations and unpleasant odor and taste may deter its use in some studies [124-126]. Oil-based solvents offer advantages such as stable drug formulations [127]. However, their low, but not necessarily absent, toxicity depends on the specific oil and route of administration [127, 128]. Additionally, high viscosity can hinder injection accuracy.

Gavage feeding provides another route for directly administering treatment to mouse pups. While it offers precise dosing control, the technique can pose risks. Studies have shown potential for intra-esophageal irritation or injury, particularly in fragile pre-weaned pups [129]. This necessitates careful technique and consideration of alternative routes when appropriate. Understanding the advantages and limitations of each administration route is crucial for designing ELI experiments. The chosen route can influence not only the well-being of the animals, but also the interpretation of the results.

**4. Animal husbandry**: While standard mouse husbandry practices—such as maintaining consistent light-dark cycles, stable room temperature, and minimal disturbance—are essential, additional considerations are necessary for studying developmental traits, particularly sexual maturation. These extra measures ensure accuracy, reliability, and scientific rigor in research. The impact of adult odor cues on development, including sexual maturation and lifespan regulation in female mice, is significant [101-107]. To mitigate unnecessary odor influences, several strategies can be implemented. Firstly, separating sires from breeding cages upon pregnancy confirmation reduces the risk of pup cannibalism and eliminates the influence of adult male odor during lactation. Secondly, while cage changes are necessary to prevent environmental complexity, they can induce stress responses in rodents. Given the well-documented link between early-life stress and long-term health in humans and animal models [6, 7, 130], a balancing act is necessary when determining cage cleaning frequency. While minimizing disruptions to minimize stress, regular changes are still required to mitigate the influence of adult urine-derived olfactory cues. Gentle handling during cleaning, standardized procedures, and uniform bedding materials aid in controlling this influence. Partial bedding changes and introducing familiar enrichment items into the new cage can further reduce stress [131]. Finally, employing individually ventilated cages (IVC) helps control the impact of airborne pheromones on rodent development [132], while maintaining optimal animal density aids in uniform density effects and odor control [132, 133].

Summary: It is widely acknowledged that early-life events exert a significant influence on later-life health outcomes. Emerging evidence suggests that ELIs hold promise for enhancing healthspan and lifespan. Mouse experiments not only yield preclinical insights into various ELIs, but also serve as valuable models for elucidating underlying mechanisms, aiding in the identification of optimized interventions for clinical translation. Furthermore, in addition to lifespan, healthspan-related parameters such as hormone levels, metabolic traits, and assessments of neuromuscular function are critical for evaluating the long-term effects of ELIs on aging. Importantly, the effectiveness of ELIs can vary significantly based on the timing, dosage, duration of the intervention, sex, genetic background, and housing conditions, which all pose challenges in identifying optimal conditions for their application. Experiments involving ELIs in mice must carefully account for these variables, considering the interactions among these factors.
